# Supplementary material for: Randomized Clinical Trial of How Long-Term Glutathione Supplementation Offers Protection from Oxidative Damage and Improves HbA1c in Elderly Type 2 Diabetic Patients
Source: Antioxidants (Basel). 2022 May 23;11(5):1026. doi: 10.3390/antiox11051026 (PMC9137531; doi:10.3390/antiox11051026)
Supplement: Supplementary file 1 [file antioxidants-11-01026-s001.zip › antioxidants-1714696-supplementary.pdf]

# Supplementary Figures

## Figure S1.

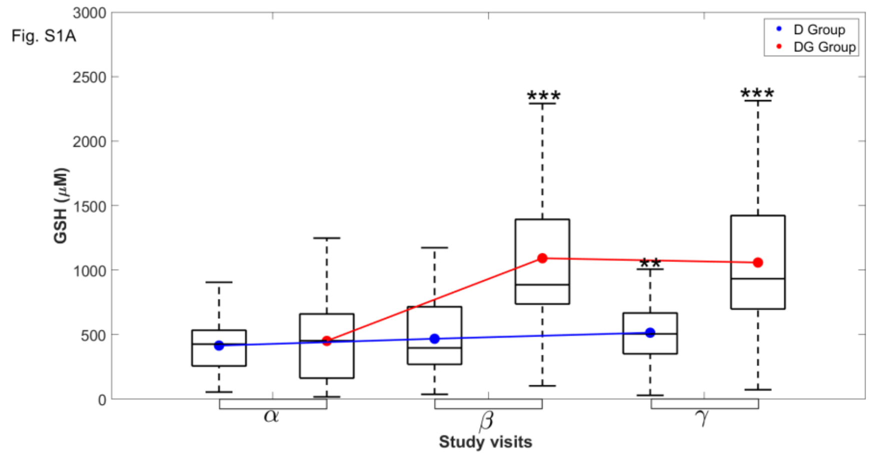


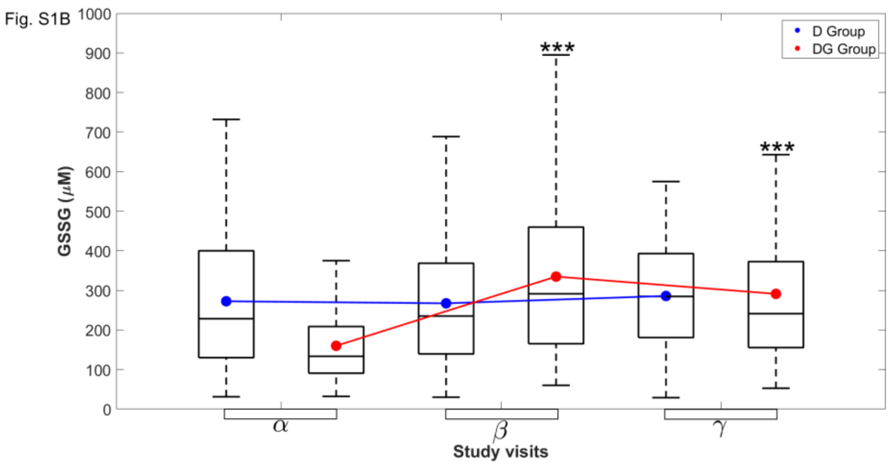

**Figure S1. Serial changes in the concentration of (A) GSH and (B) GSSG in elderly diabetic subjects.**

The measured data for A) GSH and (B) GSSG concentrations from D and DG groups in elderly subjects of D and DG groups at $\alpha$-visit ( $N_D$ =50, $N_{DG} = 53$), $\beta$-visit ( $N_D$ =50, $N_{DG} = 53$), and $\gamma$-visit ( $N_D$ = 44, $N_{DG} = 53$) are shown here with box and whiskers plots. Mean (blue for D and red for DG groups) and IQR of the data is overlaid over the corresponding box plot. The groupwise means at different visits are connected using solid lines with the same color. Significance levels (*) displayed above β, and γ visits denote the comparisons with $\alpha$ visit using permutation tests. Significance levels are *$p < 0.05$, **$p < 0.01$, ***$p < 0.001$ for respective comparisons.

**Figure S2.**

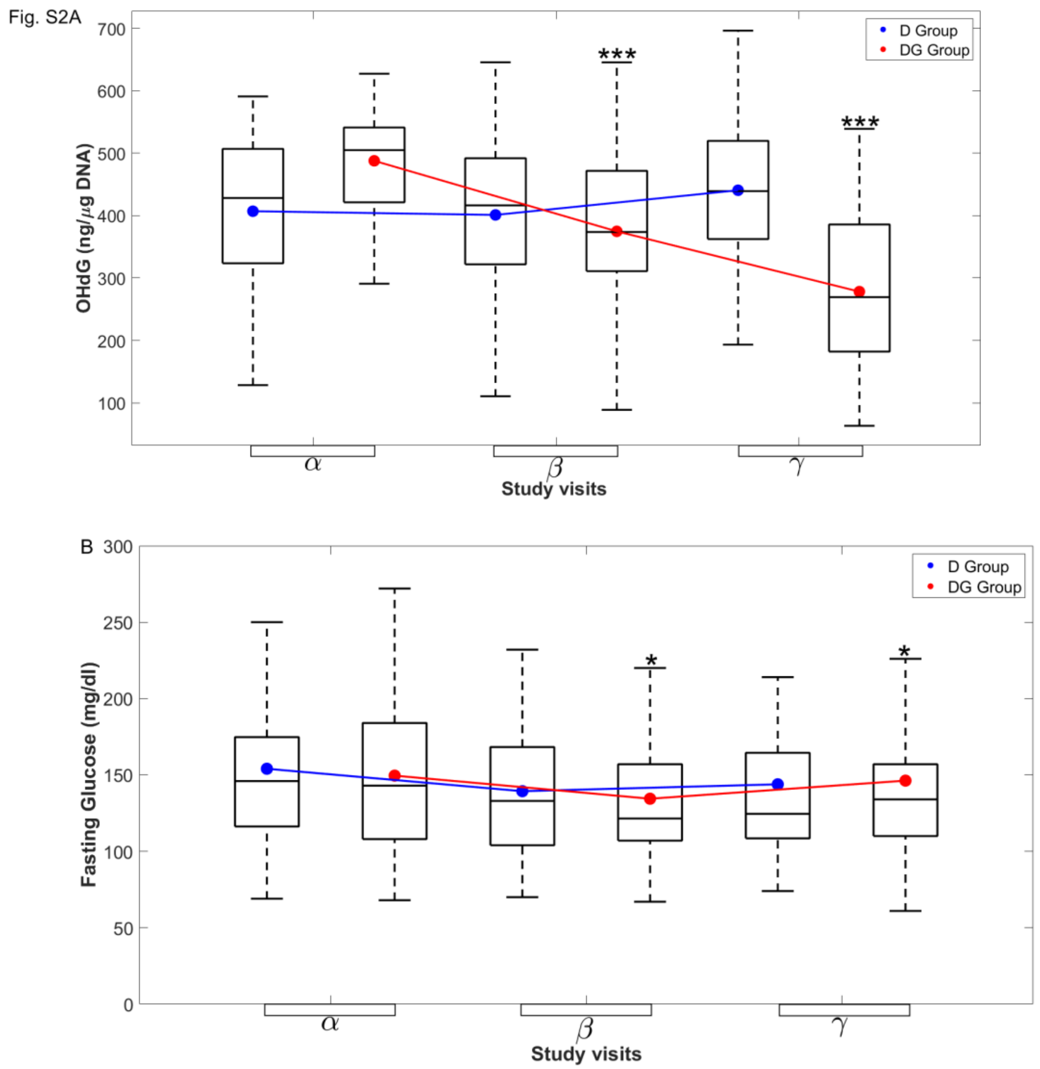
Fig. S2A
D Group
DG Group
OHdG (ng/μg DNA)
100
200
300
400
500
600
700
***
***
α
β
γ
Study visits
B
D Group
DG Group
Fasting Glucose (mg/dl)
0
50
100
150
200
250
300
*
*
α
β
γ
Study visits

C

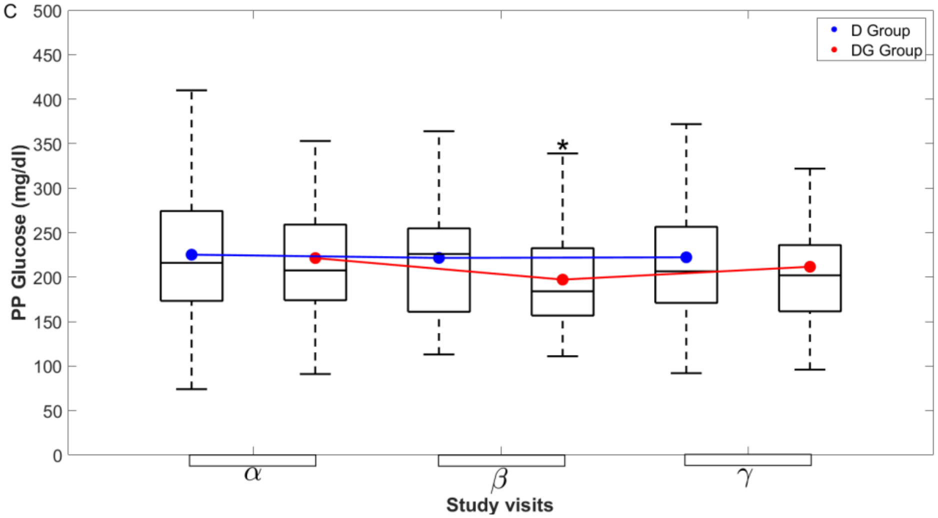

500
450
400
350
300
250
200
150
100
50
0
PP Glucose (mg/dl)
D Group
DG Group
*
α
β
γ
Study visits


D

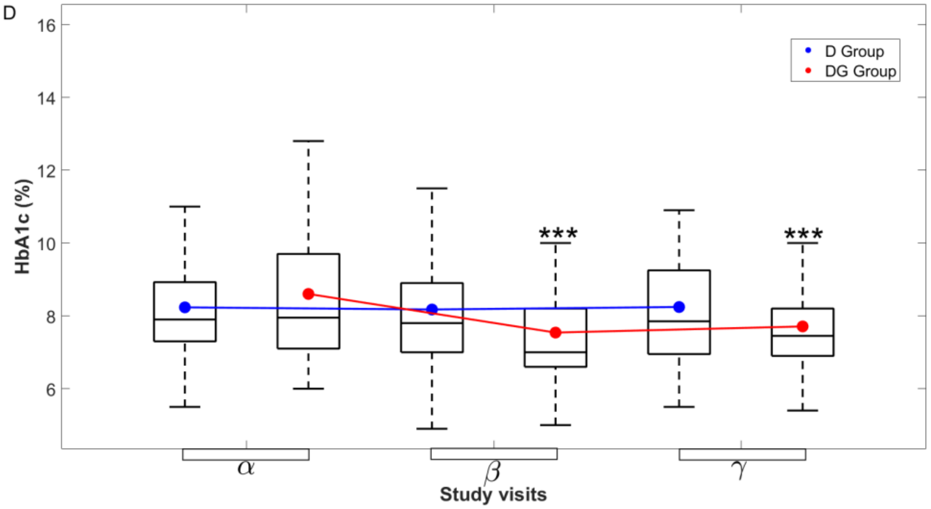

16
14
12
10
8
6
HbA1c (%)
D Group
DG Group
***
***
α
β
γ
Study visits

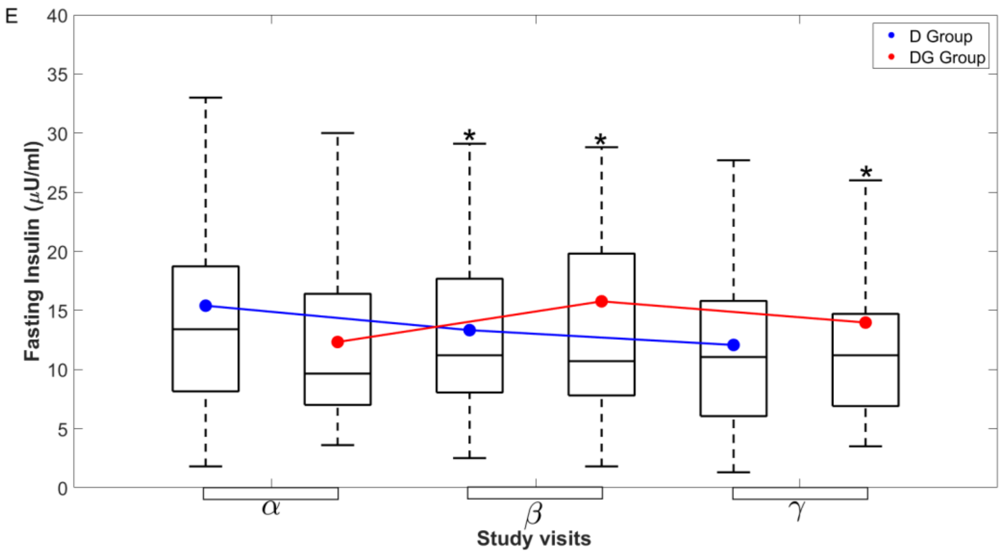
E
D Group
DG Group
Fasting Insulin ($\mu$U/ml)
0
5
10
15
20
25
30
35
40
*
*
*
$\alpha$
$\beta$
$\gamma$
Study visits

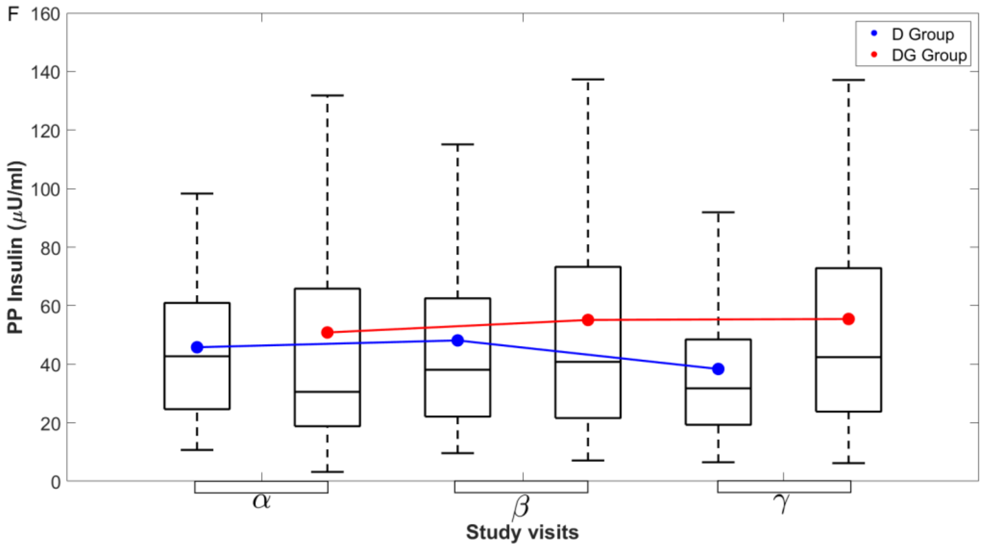
F
D Group
DG Group
PP Insulin ($\mu$U/ml)
0
20
40
60
80
100
120
140
160
$\alpha$
$\beta$
$\gamma$
Study visits

**Figure S2. Longitudinal changes in the concentration of biochemical parameters (A) 8-OHdG, (B) Fasting Glucose, (C) PP Glucose, (D) HbA1c, (E) Fasting Insulin, and (F) PP Insulin in elderly diabetic subjects.**

The measured data for (A) 8-OHdG, (B) Fasting Glucose, (C) PP Glucose, (D) HbA1c, (E) Fasting Insulin, and (F) PP Insulin concentrations in elderly subjects of D and DG groups at $\alpha$-visit (sample sizes : ( $N_D$ =51, $N_{DG} = 54$), $\beta$-visit (sample sizes for FPG, FPI, HbA1c: $N_D$ =51, $N_{DG} = 54$), for PPG: $N_D$ = 50, $N_{DG} = 53$ and for PPI: $N_D$ =50, $N_{DG} = 53$), and $\gamma$-visit (for FPG, FPI, HbA1c: $N_D$=44, $N_{DG} = 54$ , and for PPG, PPI: $N_D$ = 44, $N_{DG} = 53$ ) are shown here with box and whiskers plots. Mean (blue for D and red for DG groups) and IQR of the data is overlaid over the corresponding box plot. The groupwise means at different visits of a group are connected using solid lines with the same color. Significance levels (*) displayed above β, and γ visits denote the comparisons with $\alpha$ visit using permutation tests. Significance levels are *p < 0.05, **p < 0.01, ***p < 0.001 for respective comparisons.

**Figure S3**

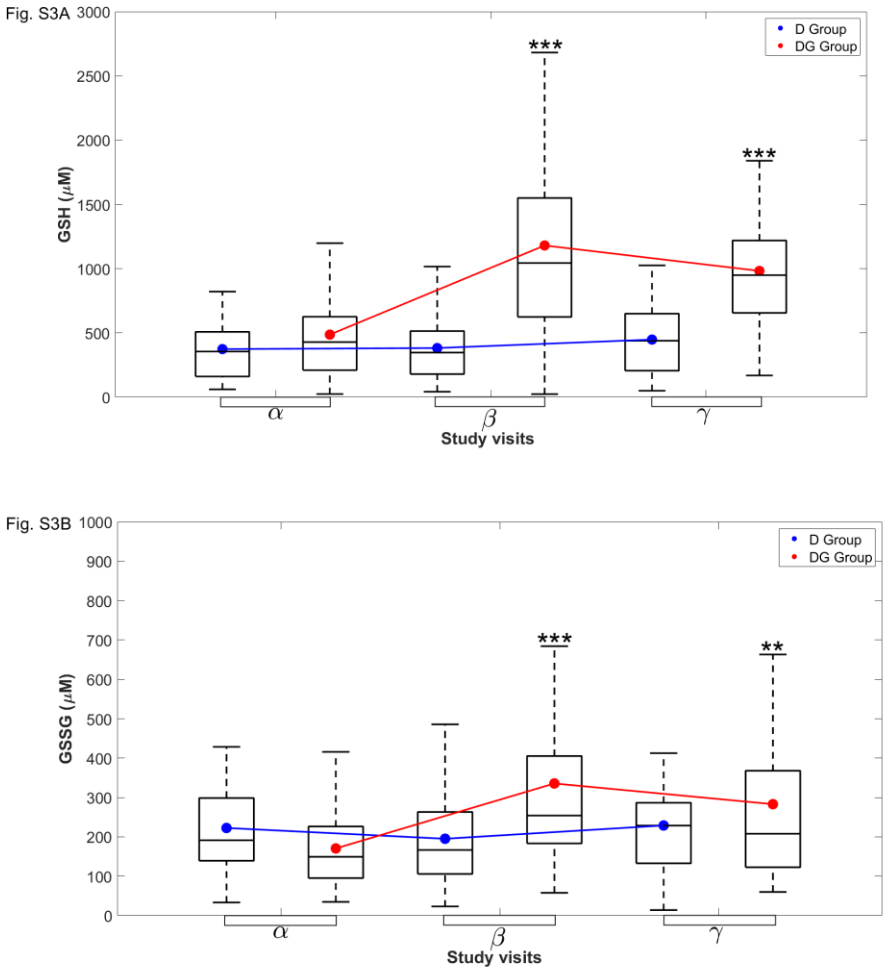


**Figure S3 Serial changes in the concentration of (A) GSH and (B) GSSG in diabetic subjects younger than 55 years.**
The measured data for A) GSH and (B) GSSG concentrations from D and DG groups in younger subjects of D and DG groups at $\alpha$-visit

( $N_D$ =43, $N_{DG} = 46$), $\beta$-visit ( $N_D$ =43, $N_{DG} = 46$), and $\gamma$-visit ( $N_D$ =43, $N_{DG} = 46$) are shown here with box and whiskers plots. Mean (blue for D and red for DG groups) and IQR of the data is overlaid over the corresponding box plot. The groupwise means at different visits are connected using solid lines with the same color. Significance levels (*) displayed above β, and γ visits denote the comparisons with $\alpha$ visit using permutation tests. Significance levels are *$p < 0.05$, **$p < 0.01$, ***$p < 0.001$ for respective comparisons.

**Figure S4**

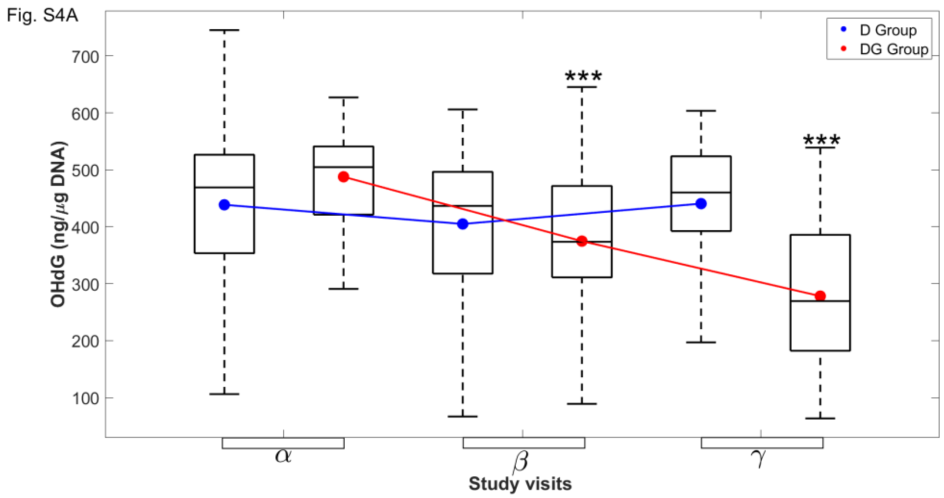
Fig. S4A
OHdG (ng/μg DNA)
700
600
500
400
300
200
100
D Group
DG Group
***
***
α
β
γ
Study visits

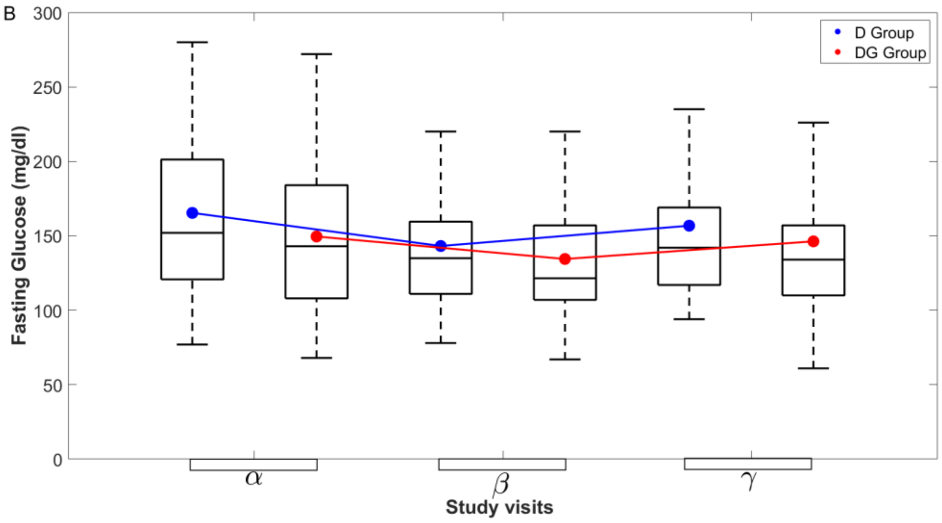
B
Fasting Glucose (mg/dl)
300
250
200
150
100
50
0
D Group
DG Group
α
β
γ
Study visits

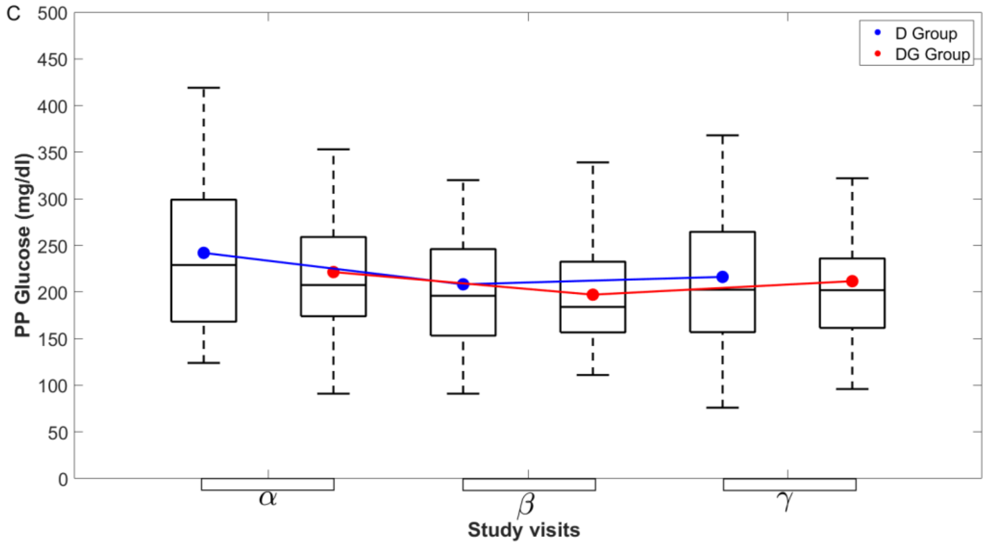
C
PP Glucose (mg/dl)
D Group
DG Group
α
β
γ
Study visits

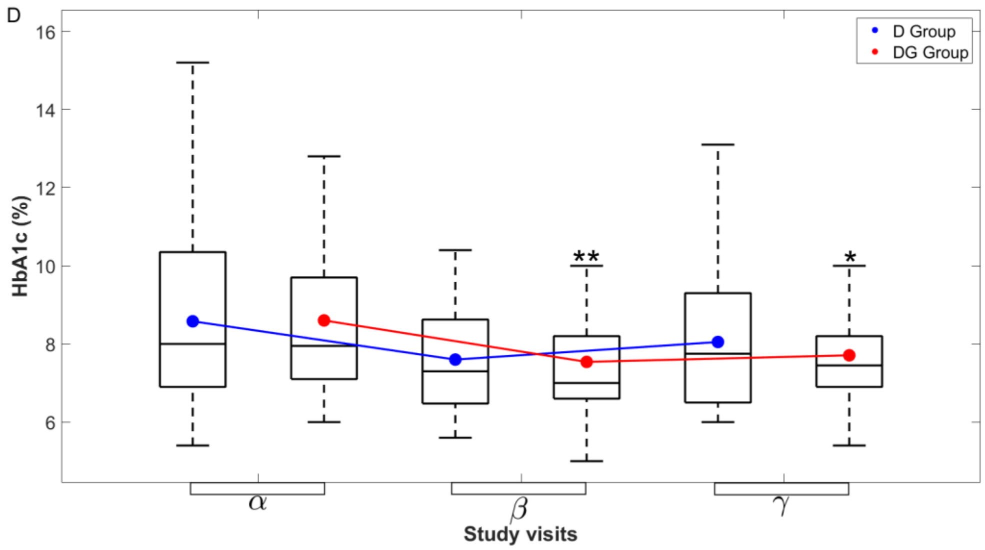
D
HbA1c (%)
D Group
DG Group
**
*
α
β
γ
Study visits

E

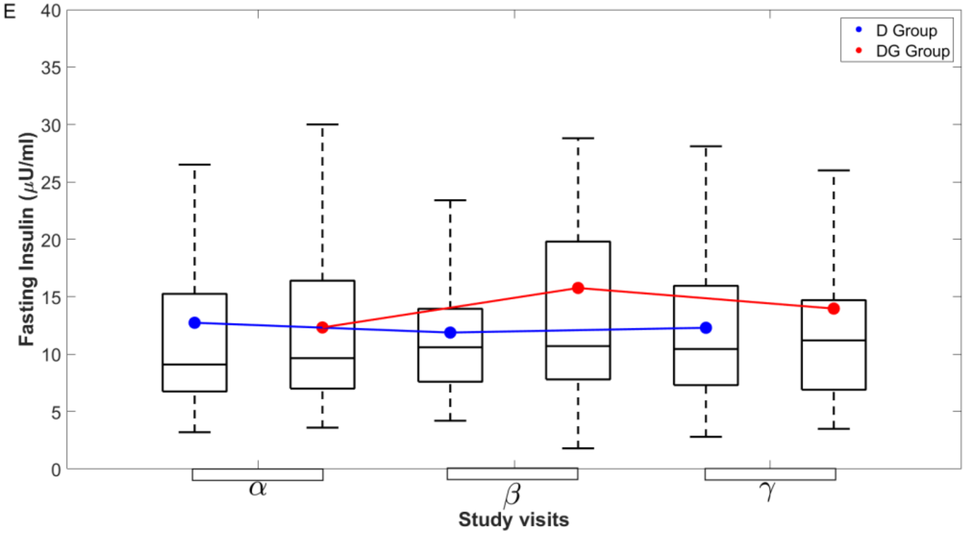

D Group
DG Group
Fasting Insulin ($\mu$U/ml)
0
5
10
15
20
25
30
35
40
$\alpha$
$\beta$
$\gamma$
Study visits


F

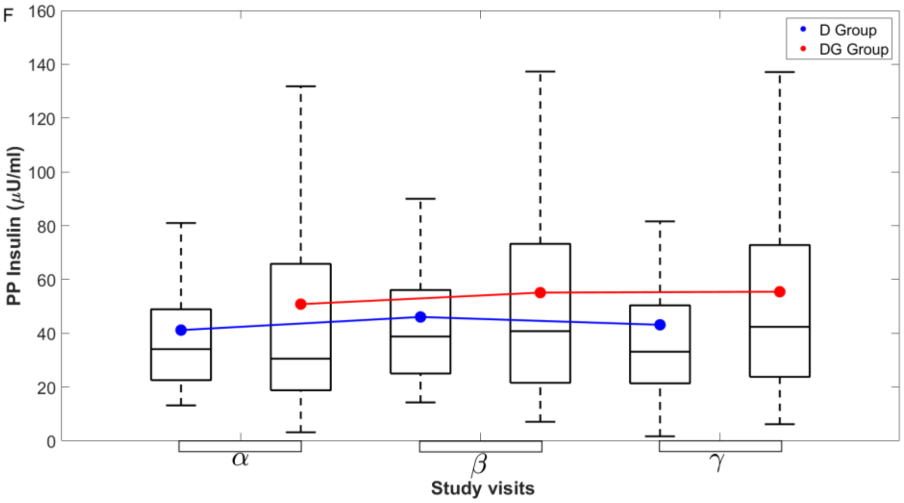

D Group
DG Group
PP Insulin ($\mu$U/ml)
0
20
40
60
80
100
120
140
160
$\alpha$
$\beta$
$\gamma$
Study visits

**Figure S4 Longitudinal changes in the concentration of biochemical parameters (A) 8-OHdG, (B) Fasting Glucose, (C) PP Glucose, (D) HbA1c, (E) Fasting Insulin, and (F) PP Insulin in subjects younger than 55 years of age**

The measured data for (A) 8-OHdG, (B) Fasting Glucose, (C) PP Glucose, (D) HbA1c, (E) Fasting Insulin, and (F PP Insulin concentrations in younger subjects of D and DG groups at $\alpha$-visit (sample sizes : ( $N_D$ = 44, $N_{DG} = 48$), $\beta$-visit (sample sizes for FPG, FPI, HbA1c: $N_D$ = 44, $N_{DG} = 48$), for PPG and for PPI : $N_D$ =44, $N_{DG} = 47$), and $\gamma$-visit (for FPG, FPI, HbA1c: $N_D$=44, $N_{DG} = 48$ , and for PPG, PPI: $N_D$ = 44, $N_{DG} = 47$ ) are shown here with box and whiskers plots. Mean (blue for D and red for DG groups) and IQR of the data is overlaid over the corresponding box plot. The groupwise means at different visits of a group are connected using solid lines with the same color. Significance levels (*) displayed above β, and γ visits denote the comparisons with $\alpha$ visit using permutation tests. Significance levels are *p < 0.05, **p < 0.01, ***p < 0.001 for respective comparisons.

**Figure S5.**

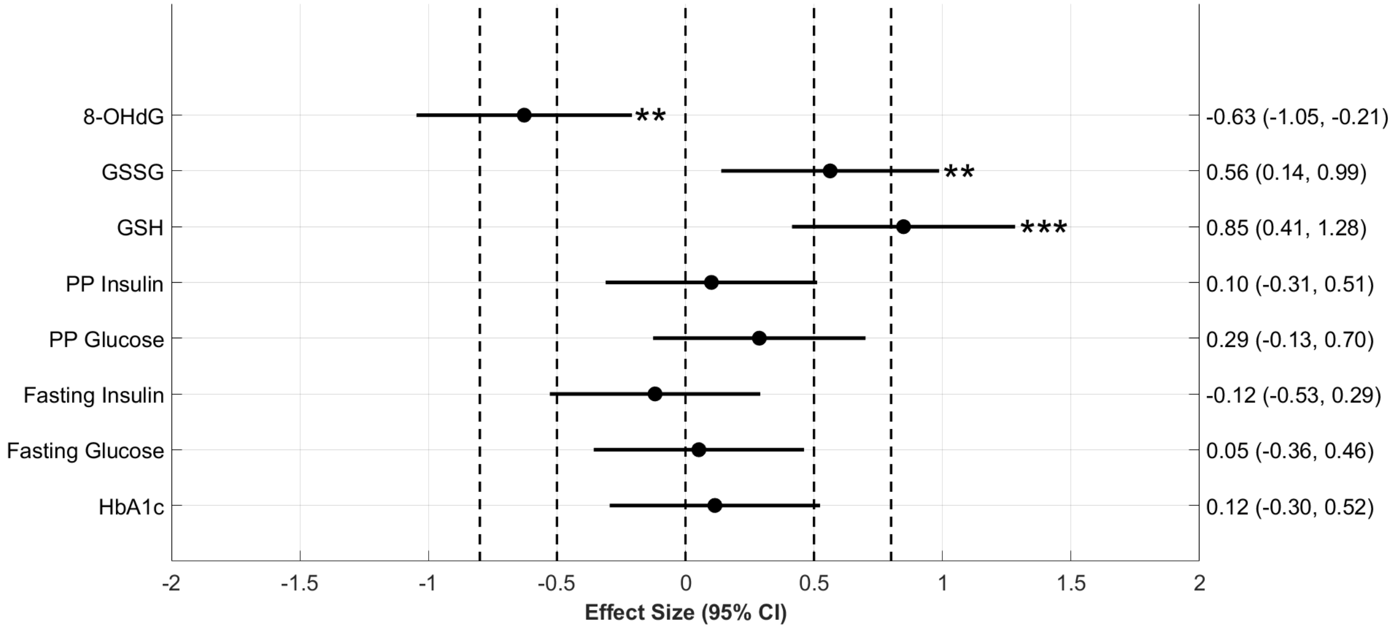


**Figure S5. The effect size of changes in blood biochemical parameters of subjects younger than 55 years of age.**
6-month changes in the biochemical parameters in younger sub-groups of D and DG were compared here on a forest plot with effect size and 95% confidence intervals. Effect size (Cohen's d) calculated between 6-month changes in the concentration of biochemical variables, are denoted on the x-axis. The group-wise means of 6-month changes in the concentration of these variables were compared using two -sample permutation tests. The significance of these comparisons are denoted with the p values mentioned to the right of horizontal lines for CI. Significance levels are *p < 0.05, **p < 0.01, ***p < 0.001 for respective comparisons.

**Figure S6A.**

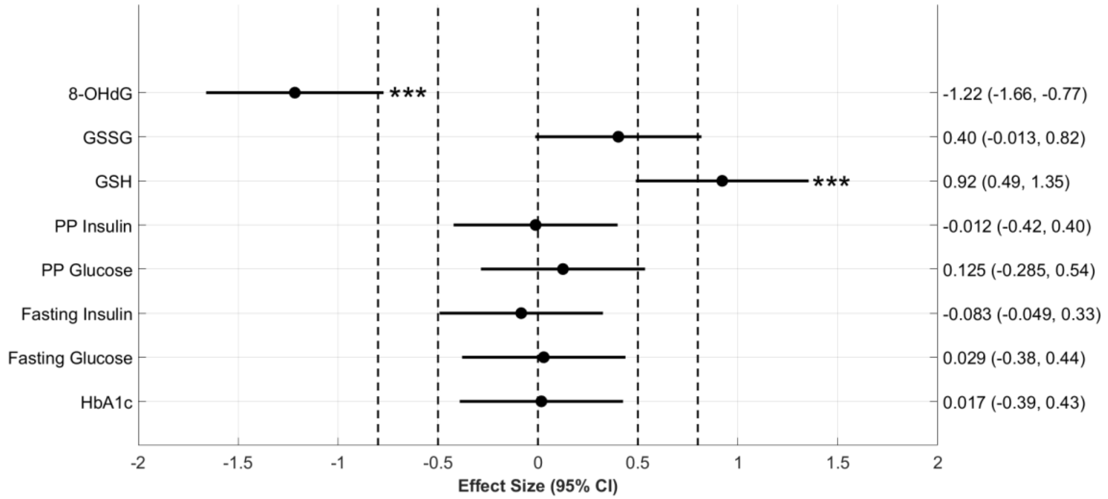


**Figure S6B**

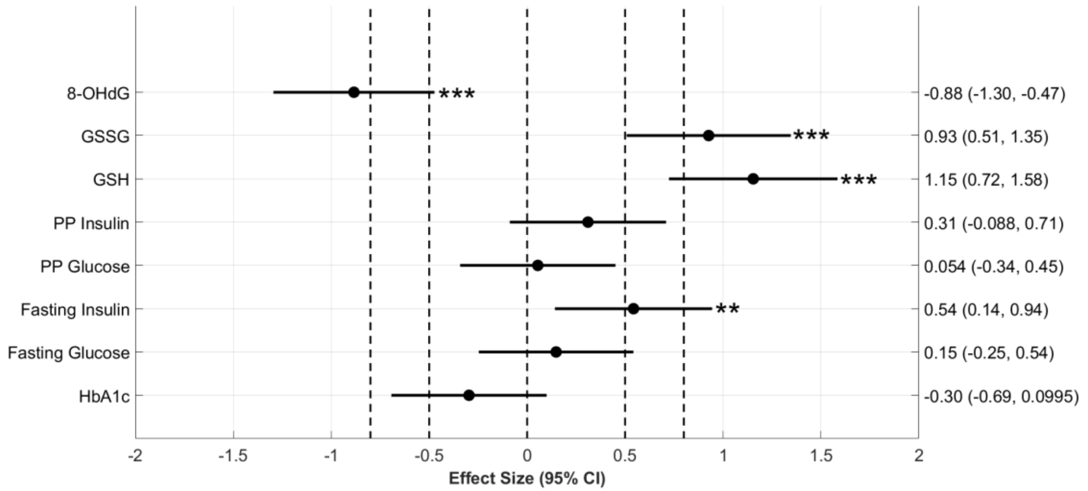

**Figure S6. The effect size of changes in blood biochemical parameters in (A) females, (B) males in the study**

6-month changes in the biochemical parameters in female subgroups in D and DG (A), male subgroups in D and DG (B) were compared here on a forest plot with effect size and 95% confidence intervals. Effect size (Cohen's d) calculated between 6-month changes in the concentration of biochemical variables, are denoted on the x-axis. The group-wise means of 6-month changes in the concentration of these variables were compared using two-sample permutation tests. The significance of these comparisons are denoted with the p values mentioned to the right of horizontal lines for CI. Significance levels are *$p < 0.05$, **$p < 0.01$, ***$p < 0.001$ for respective comparisons.

**Figure S7A.**

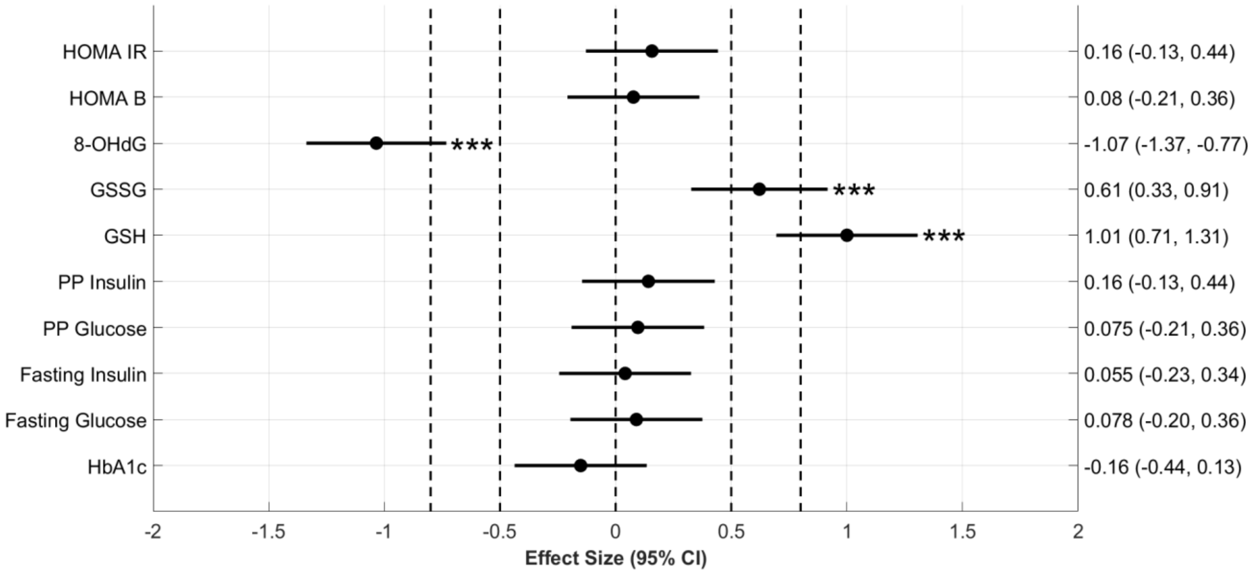


**Figure S7B.**

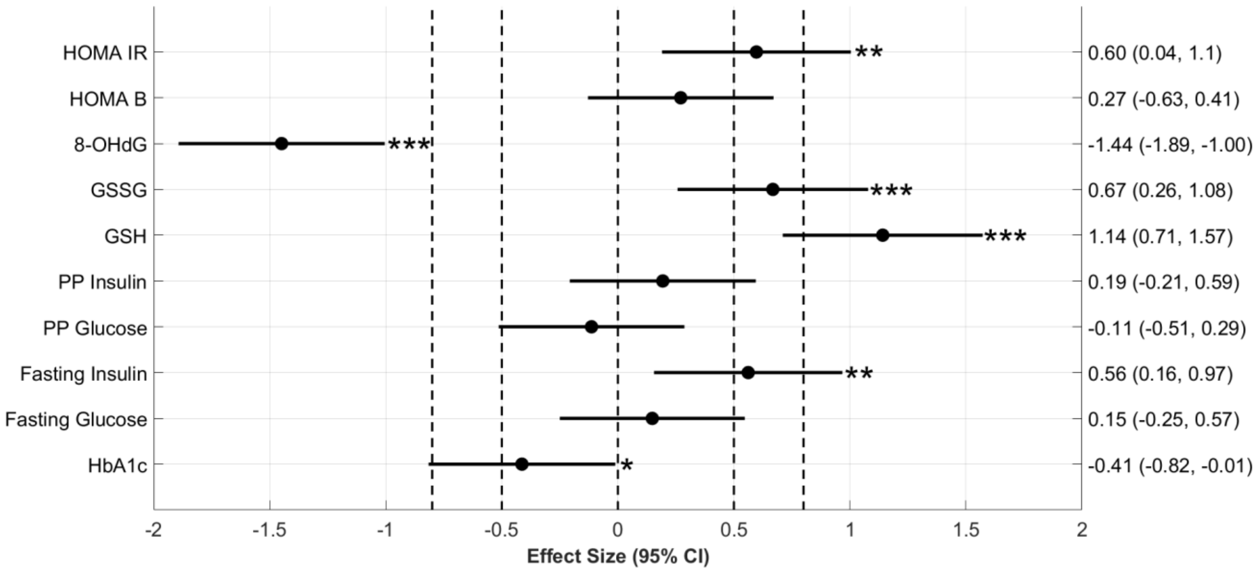


**Figure S7. The effect size of changes in blood biochemical parameters, HOMA IR and HOMA β (A) between D and DG groups and (B) elder subgroups of D and DG.**

6-month changes in the biochemical parameters, HOMA IR and HOMA β were compared here (A) between D and DG groups and (B) elderly subgroups of D and DG groups on a forest plot with effect size and 95% confidence intervals. Effect size (Cohen's d) calculated between 6-month changes in the concentration of biochemical variables, are denoted on the x-axis. The group-wise means of 6-month changes in the concentration of these variables were compared using two-sample permutation tests. The significance of these comparisons are denoted with the p values mentioned to the right of horizontal lines for CI. Significance levels are *$p < 0.05$, **$p < 0.01$, ***$p < 0.001$ for respective comparisons.

**Supplementary Tables**

**Table S1**

| | Study | Number of individuals | Dose & duration | Results and Conclusion |
|---|---|---|---|---|
| 1 | Pailisso et al, 1992a | 10 diabetic<br>10 healthy | GSH infusion 15 mg per minute for 60 minutes | Plasma GSH/GSSG ratio significantly improved total body glucose disposal in healthy subjects and in diabetic patients. In diabetic patients GSH infusion potentiated the beta-cell response to glucose slightly. The plasma GSH/GSSG ratio seems to play a major role in the modulation of glucose homeostasis mainly in diabetics. |
| 2 | Pailisso et al, 1992b | 10 Elderly healthy<br>10 Elderly with impaired glucose tolerance | GSH infusion 10 mg per minute for 120 min | In subjects with normal glucose tolerance, glutathione infusion failed to affect β-cell response to glucose. In contrast, glutathione significantly potentiated glucose-induced insulin secretion in patients with impaired glucose tolerance (IGT). Glutathione infusion enhances insulin secretion in elderly people with IGT. |
| 3 | Sekhar et al, 2011a | 8 elderly healthy | Oral supplementation of cystine (0.81mmol per kg per day) and glycine (1.33 mmol per kg per day for 14 days) | Dietary supplementation of cysteine and glycine restores glutathione synthesis and concentrations and lowers levels of oxidative stress and oxidant damages in elderly human subjects. |
| 4 | Sekhar et al, 2011b | 12 diabetic<br>12 healthy | Oral treatment of cystine 0.81mmol per kg per day and glycine 1.33 mmol per kg per day for 14 days | Dietary supplementation with GSH precursor amino acids glycine restored GSH synthesis and lower oxidative stress and oxidant damage in diabetic individuals in the face of persistent hyperglycaemia |

| | | | | |
|---|---|---|---|---|
| 5 | Allen and Bradley, 2011 | 40 healthy | Oral GSH supplementation<br>500 mg twice daily for 4 weeks | Short-term, oral intake of GSH does not improve glutathione status, nor reduce markers of oxidative stress in healthy adults. |
| 6 | Richie et al, 2015 | 54 healthy non-smokers | Oral GSH supplementation with either 250 mg or 1000 mg GSH per day for 1,3 and 6 months | GSH supplementation was effective at increasing body compartment stores of GSH. In most cases, the increase was dose and time dependent, and levels returned to baseline after a 1 month washout period. |
| 7 | Schmitt et al, 2015 | 20 volunteers with metabolic syndrome | NAC, oral GSH and subligual GSH<br>Oral NAC contains 200 mg NAC<br>Oral GSH 150 mg per tab 3tab/day<br>Oral subligual GSH-3 tab/day 1 tab contains 150 GSH for three weeks | Compared to oral GSH group, an increase in total and reduced GSH levels in plasma and a higher GSH/GSSG ratio was observed in sublingual GSH group. After 3 weeks of administration, there was a significant increase of vitamin E level in plasma only in sublingual GSH group. Results demonstrate the superiority of a new sublingual form of GSH over the oral GSH form and NAC in terms of GSH supplementation. |
| 8 | Buonocore et al, 2016 | 15 healthy | Orobuccal fast slow oral formulation single tablet containing<br>250 mg GSH<br>50 mg cystine<br>40 mg cystine<br>55mcg selenium<br>For 0 , 30 and 60 minute | The intake of GSH formulated through optimized oro-buccal fast-slow release tablets led to increase in blood GSH concentration. |
| 9 | Sinha et al, 2018 | 12 healthy | Liposomal GSH at 500 and 1000 mg per day for 1, 2 and 4 weeks | Daily liposomal GSH supplementation elevated body stores of GSH and impacted immune function and levels of oxidative stress. Overall, there were no differences observed between two |

| | | | | |
|---|---|---|---|---|
| | | | | groups, but statistical power was limited due to the small sample size in this study. |
| 10 | Bruggman et al, 2019 | 15 healthy | Orobuccal non liposomal nano sized glutathione 200 mg/ml administered orally for oral 0, 5, 10, 30, 60 and 120 minutes | Non-liposomal nano-sized glutathione solution increased multiple blood glutathione parameters compared to placebo. |

**Table S1: Summary of Clinical trials conducted using GSH/different forms of GSH/precursors of GSH.**

**Table S2.**

A

| **Sr No.** | **Type of treatment in Diabetic (D) group** | **No. of subjects** |
|---|---|---|
| 1 | Biguanides | 19 |
| 2 | Sulphonylureas | 10 |
| 3 | Thiazolidinediones | 1 |
| 4 | Meglitinides | 1 |
| 5 | Biguanides & Sulphonylureas | 35 |
| 6 | Biguanides & DPP-4 inhibitor | 6 |

| | | |
|---|---|---|
| 7 | Biguanides & Insulin | 2 |
| 8 | Sulphonylureas & glucosidases inhibitor | 1 |
| 9 | Biguanides & DPP-4 inhibitor | 4 |
| 11 | DPP-4 inhibitor & Sulphonylureas | 1 |
| 12 | Biguanides & Sulphonylureas &Thiazolidinediones | 2 |
| 13 | Biguanides & Sulphonylureas & DPP-4 inhibitor | 2 |
| 14 | Biguanides & Sulphonylureas & Insulin | 1 |
| 15 | Biguanides & Sulphonylureas & α-glucosidases inhibitor | 5 |
| 16 | Biguanides & DPP-4 inhibitor & α-glucosidases inhibitor | 1 |
| 17 | Controlled by exercise | 10 |
| | Total | 101 |

B

| Sr No. | Type of treatment Diabetic with Glutathione (DG) group | No. of subjects |
|---|---|---|
| 1 | Biguanides | 35 |
| 2 | Sulphonylureas | 2 |
| 3 | Insulin | 1 |
| 4 | Biguanides & Sulphonylureas | 26 |
| 5 | Biguanides & DPP-4 inhibitor | 7 |
| 6 | Biguanides & Insulin | 1 |
| 7 | Biguanides & α-glucosidases inhibitor | 1 |
| 8 | DPP-4 inhibitor & α-glucosidases inhibitor | 1 |
| 9 | DPP-4 inhibitor & Insulin | 1 |
| 10 | Biguanides & Sulphonylureas &Thiazolidinediones | 1 |
| 11 | Biguanides & Sulphonylureas & DPP-4 inhibitor | 11 |
| 12 | Biguanides & Sulphonylureas & Insulin | 1 |
| 13 | Biguanides & Sulphonylureas & α-glucosidases inhibitor | 1 |

| | | |
|---|---|---|
| 14 | Biguanides & α-glucosidases inhibitor & DPP-4 inhibitor | 1 |
| 15 | Controlled by exercise | 5 |
| | Total | 95 |

**Table S2. Anti-diabetic treatment in D (A) and DG (B) groups.** Number of subjects in D and DG groups with different types of anti-diabetic treatment are listed here.

**Table S3.**

| **Groups** | **Variables** | ***α*-Visit**<br>--------<br>Mean (95% CI) | ***β*-Visit**<br>--------<br>Mean (95% CI) | **γ-Visit**<br>--------<br>Mean (95% CI) |
|---|---|---|---|---|
| **Control** | **HbA1c** (%) | 5.6 (5.5-5.6) | 5.6 (5.5-5.7) | 5.7 (5.6-5.7) *** |
| | **FPG** (mg/dl) | 91 (89-92) | 91 (89-92) | 91 (90-93) |
| | **PPG** (mg/dl) | 108 (105-112) | 105 (101-109) * | 108 (102-115) |
| | **FPI** (µU/ml) | 10.7 (9.5-11.8) | 12 (10.1-13.9) | 11.8 (10.9-12.8) * |
| | **PPI** (µU/ml) | 51.5 (42.6-60.4) | 55.9 (45.6-66.2) | 52.3 (41.3-63.3) |
| | **GSH** (µM) | 842 (758-926) | 868 (774-962) | 861 (770-952) |
| | **GSSG** (µM) | 218 (193-244) | 247 (219-276) | 226 (197-256) |

| | | | | |
|---|---|---|---|---|
| **D** | **HbA1c** (%) | 8.4 (8.1-8.8) | 7.9 (7.6-8.2) ** | 8.2 (7.8-8.6) |
| | **FPG** (mg/dl) | 160 (148-172) | 143 (134-152) ** | 151 (139-163) |
| | **PPG** (mg/dl) | 234 (217-250) | 217 (203-231) | 220 (203-238) |
| | **FPI** (µU/ml) | 14.2 (12.1-16.2) | 12.7 (11.4-14.0) * | 12.1 (10.5-13.7) * |
| | **PPI** (µU/ml) | 43.4 (38.1-48.7) | 47 (40.5-53.6) | 40.5 (34.2-46.7) |
| | **GSH** (µM) | 395 (351-440) | 428 (376-480) | 484 (430-537) *** |
| | **GSSG** (µM) | 249 (220-279) | 236 (205-268) | 262 (233-291) |
| **DG** | **HbA1c** (%) | 8.5 (8.1-8.9) | 7.7 (7.4-8) *** | 7.9 (7.6-8.2) *** |
| | **FPG** (mg/dl) | 153 (141-164) | 141 (133-150) | 150 (138-161) |
| | **PPG** (mg/dl) | 222 (207-237) | 211 (195-227) | 218 (202-234) |
| | **FPI** (µU/ml) | 14.6 (10.2-18.9) | 14.6 (11.9-17.3) | 13.9 (11.8-15.9) |
| | **PPI** (µU/ml) | 48.4 (39.1-57.6) | 49.5 (41.7-57.3) | 52.3 (43.7-60.9) |
| | **GSH** (µM) | 465 (395-534) | 1126 (994-1258) *** | 1022 (919-1124) *** |
| | **GSSG** (µM) | 163 (142-183) | 333 (290-375) *** | 286 (246-326) *** |

**Table S3. Biochemical measurements at different visits in Control, D, and DG groups.** Biochemical measurements of subjects in Control, D, and DG groups at $\alpha$, $\beta$, and $\gamma$-visits are given herewith mean and 95% confidence interval. Significance levels (*) displayed at $\beta$ and $\gamma$-visits denote the comparisons with $\alpha$-visit using permutation tests. Significance levels are *p < 0.05, **p < 0.01, ***p < 0.001 for respective comparisons (See Table S6 for p-values of these comparisons using permutation tests and alternate comparisons using t-tests).

**Table S4.**

| Variables | Control versus D (p-value) | | Control versus DG (p-value) | | D versus DG (p-value) | |
|---|---|---|---|---|---|---|
| | Permutation test | t-test | Permutation test | t-test | Permutation test | t-test |
| **Age** | $1.41\times 10^{-16}$*** | $3.6\times 10^{-17}$*** | $7.96\times 10^{-18}$*** | $2.12\times 10^{-18}$*** | 0.77 | 0.77 |
| **BMI** | 0.88 | 0.88 | 0.10 | 0.10 | 0.071 | 0.073 |
| **HbA1c** | $4.12\times 10^{-46}$*** | $3.79\times 10^{-35}$*** | $8.41\times 10^{-55}$*** | $1.36\times 10^{-35}$*** | 0.72 | 0.72 |
| **FPG** | $2.89\times 10^{-36}$*** | $9.92\times 10^{-24}$*** | $8.18\times 10^{-32}$*** | $2.26\times 10^{-21}$*** | 0.38 | 0.38 |
| **FPI** | 0.002** | 0.003 ** | 0.029* | 0.086 | 0.93 | 0.88 |
| **PPG** | $1.9\times 10^{-44}$*** | $2.23\times 10^{-34}$*** | $4.32\times 10^{-45}$*** | $6.5\times 10^{-34}$*** | 0.302 | 0.30 |
| **PPI** | 0.13 | 0.13 | 0.63 | 0.63 | 0.37 | 0.36 |
| **GSH** | $7.51\times 10^{-15}$*** | $4.94\times 10^{-17}$*** | $3.57\times 10^{-11}$*** | $9.23\times 10^{-11}$*** | 0.098 | 0.098 |

| | | | | | | |
|---|---|---|---|---|---|---|
| **GSSG** | 0.12 | 0.12 | $8.33\times 10^{-4}$*** | $8.80\times 10^{-4}$*** | $2.99\times 10^{-5}$*** | $3.74\times 10^{-6}$*** |

**Table S4. Inter-group comparisons of baseline characteristics.** Variables at the α visit in different groups were compared using two sample permutation tests and t-tests. p-values of comparison are shown here in this table. Significance levels are *p < 0.05, **p < 0.01, ***p < 0.001 for respective comparisons.
